# Fulfillment status of hypertriglyceridemia and hypofibrinogenemia in children with hemophagocytic lymphohistiocytosis and risks of multiple organ dysfunction syndrome and early mortality

**DOI:** 10.1186/s13023-022-02315-8

**Published:** 2022-04-11

**Authors:** Xun Li, Haipeng Yan, Ting Luo, Zhenghui Xiao, Ling Gong, Jiaotian Huang, Xinping Zhang, Mincui Zheng, Zhenya Yao, Ping Zang, Desheng Zhu, Xiulan Lu

**Affiliations:** 1 Department of Pediatric Intensive Care Unit (PICU), Pediatrics Research Institute of Hunan Province, Hunan Children’s Hospital, Changsha, China; 2 Department of Pediatric Intensive Care Unit (PICU), Hunan Children’s Hospital, Changsha, China; 3 Department of Pediatric Hematology, Hunan Children’s Hospital, Changsha, China

**Keywords:** Child, Fibrinogen, Multiple organ dysfunction syndrome, Hemophagocytic lymphohistiocytosis, Triglyceride

## Abstract

**Background:**

Hemophagocytic lymphohistiocytosis (HLH) is a life-threatening disorder. How to stratify high risk patients is one of the current challenges for the treatment of HLH. HLH patients usually fulfill multiple but not all eight diagnostic criteria. Different combinations of the fulfilled criteria may naturally cluster into previously undescribed subsets or phenotypes that may have different pathophysiology and demonstrate different risks for a poor outcome. The objectives of this study were to identify HLH subgroups according to the fulfillment of diagnostic criteria and evaluate the risk of multiple organ dysfunction syndrome (MODS) and 30-day mortality for subgroups. We retrospectively collect medical records of patients with discharge diagnosis of HLH between June 2015 and October 2018 from a tertiary children’s hospital in China. Latent class analysis was used to identify class defining variables from HLH diagnostic items, and subgroups were defined according to different combinations of the class defining variables.

**Results:**

Triglyceride and fibrinogen were identified as the class defining variables. When evaluated in combinations, patients with hypertriglyceridemia and normal fibrinogen levels during hospitalization had the lowest risks for MODS (27.8%, OR = 1) and 30-day mortality (18.8%, OR = 1), and patients with normal triglyceride and hypofibrinogenemia had the highest risks for MODS (86.2%, OR = 16.24, *P* = 0.0002) and 30-day mortality (57.1%, OR = 5.78, *P* = 0.0187). The fulfillment status of hypertriglyceridemia and hypofibrinogenemia within 72 h of hospital admission was also associated with the risk of adverse outcomes.

**Conclusions:**

The fulfillment status of hypertriglyceridemia and hypofibrinogenemia were associated with the risks of MODS and 30-day mortality among pediatric HLH patients. Further studies are needed to validate this association and investigate its clinical utility in the severity evaluation for HLH.

**Supplementary Information:**

The online version contains supplementary material available at 10.1186/s13023-022-02315-8.

## Background

Hemophagocytic lymphohistiocytosis (HLH) comprises a heterogeneous class of disorders characterized by clinical signs and symptoms of extreme immune activation. It is a life-threatening disorder that can rapidly deteriorate and lead to multiple organ failure and death [1–3]. How to stratify high risk patients and initiate adequate treatment are the current challenges for the treatment of HLH [2, 4].

HLH has been recognized to comprise a heterogeneous spectrum of clinically similar but etiologically diverse subtypes [5]. According to HLH-2004 diagnostic criteria, the diagnosis of HLH can be established if a genetic diagnosis consistent with HLH, or five out of eight criteria fulfilled. Traditionally HLH was subdivided as primary, which is attributed to underlying genetic defects, or secondary, which is triggered by other diseases like infections, malignancy, lymphoma, or autoimmune diseases. However, evidences have shown that primary HLH could triggered by other diseases and secondary HLH may display some mutations or polymorphisms as well [6]. Besides, the typical manifestations were not distinguished from the primary or secondary classification [4]. Therefore, HLH is now been considered as a threshold disease, with genetic factors and other endogenous and exogenous factors interacting until a threshold is reached [5].

Regardless or the primary of secondary classification, HLH patients usually fulfill multiple but not all eight criteria. Single items from the criteria as prognostic factors has been explored by several studies. For example, decreased hemoglobin [7], platelet [8], and neutrophils [8], hyperferritinemia [9, 10], and hypofibrinogenemia [7] were found to be significantly associated with early death in children with HLH. However, to date no consensus exists on the interpretation of the fulfilled criteria as a whole, reflecting our limited understanding of the disease mechanism and limited ability to interpret the clinical relevance of the diagnostic criteria. Different combinations of the fulfilled criteria may naturally cluster into previously undescribed subsets or phenotypes that may have different pathophysiology and demonstrate different risks for a poor outcome. Therefore, the combination of fulfilled criteria and its association with disease severity is yet to be explored.

The objectives of this study were to identify HLH subgroups according to the fulfillment of diagnostic criteria and evaluate the risk of multiple organ dysfunction syndrome (MODS) and 30-day mortality for subgroups. We used a two-step strategy to identify HLH subgroups from the fulfillment status of diagnostic criteria: first, using latent class analysis (LCA), a statistical technique which bases on the hypothesis that the data contain several unobserved classes [11], to identify class defining variables from HLH diagnostic items; second, defining subgroups according to different combinations of the class defining variables. After the identification of subgroups, the demographic and clinical characteristics among subgroups were compared, and the risk of MODS and early death for each subgroups were estimated. The primary subgroup allocation were based on the worst values of class defining variables during hospitalization. Grouping according to the worst values within 72 h of hospital admission were also applied to explore the utility of the grouping method for the early evaluation of disease severity. Our hypothesis was that different combinations of main features of HLH could be used to evaluated disease severity and prognosis. The finding of this study should add knowledge for the interpretation HLH diagnostic criteria and provide a candidate method to evaluate disease severity.

## Results

During the study period, 145 patients were diagnosed with HLH. Among them, 28 patients were excluded from the present study because of essential diagnostic data missing. The final analysis included 117 pediatric HLH patients.

**Fig. 1:**
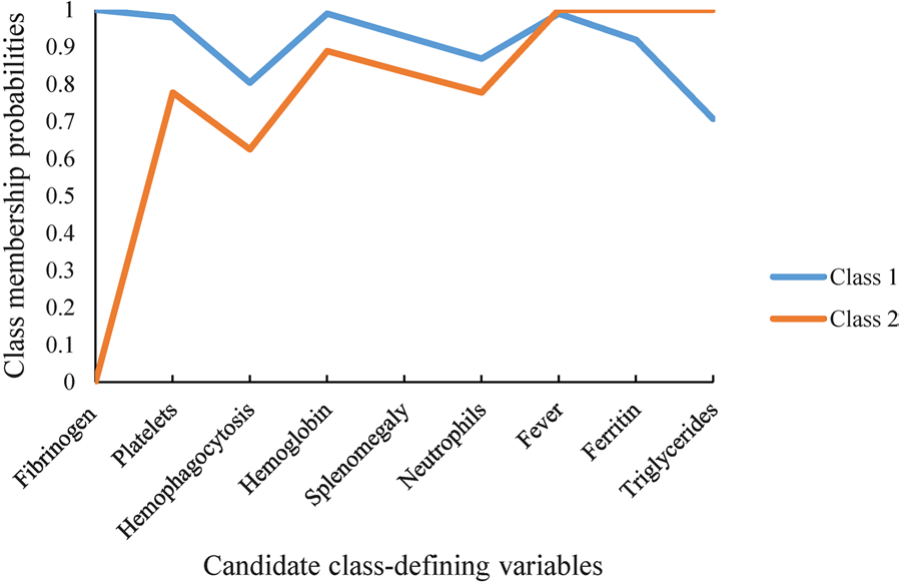
Class membership probabilities for class-predicting variables. The variables are sorted from left to right in descending order for the difference in values between Class 1 and Class 2

Nine candidate variables were applied as latent class defining variables. Additional file 1: Table S1 shows the fit statistics of three LCA models. The two-class model, with a desirable entropy and a low AIC and BIC, was judged to be the best fit for this population. The class membership probability for each model-defining variable were presented in Additional file 1: Table S2. For each variable, the difference for the class membership probabilities between Class 1 and Class 2 were calculated, and the largest differences were observed for triglyceride and fibrinogen (Fig. 1). Therefore, triglyceride (TG) and fibrinogen (Fib) were selected as the class defining variables.

Table 1 shows the distribution of the worst values of TG and Fib among the study population. Patients developed MODS and/or dead within 30 days had lower levels of triglyceride and fibrinogen (*P* < 0.05).

**Table 1:** Distribution of the worst values for triglyceride and fibrinogen among 117 pediatric HLH patients

Worst value during hospitalization	All (n = 117)	MODS			30-day survival		
		No (n = 46)	Yes (n = 71)	*P*	Survive (n = 78)	Death (n = 36)	*P*
Triglyceride, mmol/L							
Median (Q1, Q3)	**4.08 (3.01, 5.45)**	**4.30 (3.59, 6.16)**	**3.72 (2.64, 4.93)**	**0.0069**	**4.31 (3.35, 6.11)**	**3.47 (2.16, 4.41)**	**0.0028**
Min, max	**1.05, 11.41**	**1.81, 11.41**	**1.05, 11.15**		**1.81, 11.41**	**1.05, 10.44**	
< 3.0 mmol/L, n (%)	**29 (24.8)**	**4 (8.7)**	**25 (35.2)**	**0.0012**	**12 (15.4)**	**16 (44.4)**	**0.0019**
≥ 3.0 mmol/L, n (%)	**88 (75.2)**	**42 (91.3)**	**46 (64.8)**		**66 (84.6)**	**20 (55.6)**	
Fibrinogen, mg/dL							
Median (Q1, Q3)	**83 (60, 122)**	**113.50 (68, 159)**	**69 (60, 101)**	**0.0004**	**88.5 (61, 124)**	**65.5 (60, 108)**	**0.0481**
Min, max	**44, 349**	**44, 349**	**51, 256**		**44, 229**	**51, 349**	
> 150 mg/dL, n (%)	**18 (15.4)**	**13 (28.3)**	**5 (7.0)**	**0.0019**	13 (16.7)	3 (8.3)	0.3842
≤ 150 mg/dL, n (%)	**99 (84.6)**	**33 (71.7)**	**66 (93.0)**		65 (83.3)	33 (91.7)	

HLH, hemophagocytic lymphohistiocytosis; MODS, multiple organ dysfunction syndrome

Values in bold are statistically significant (*P* < 0.05)

**Fig. 2:**
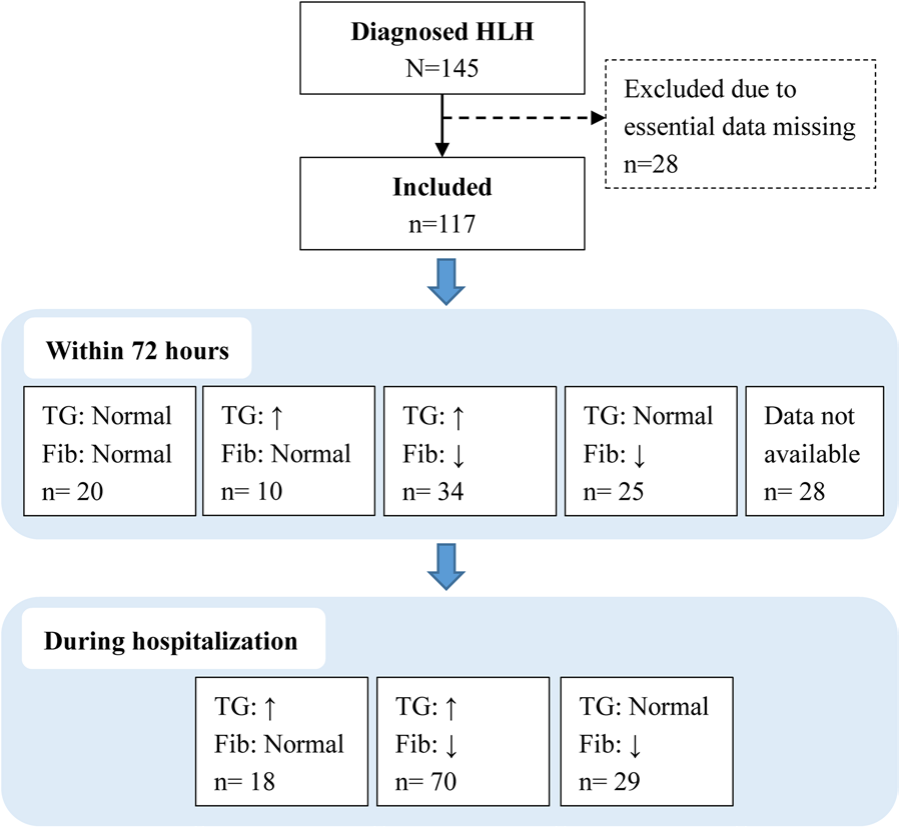
Subgroup allocation according to the worst values of triglyceride and fibrinogen. Fib, fibrinogen. HLH, hemophagocytic lymphohistiocytosis. TG, triglyceride. TG ↑, ≥ 3.0 mmol/L. Fib ↓, ≤ 150 mg/dL

Four subgroups were derived from the combination of worst value status for TG and Fib: (1) normal TG and normal FIB (TG Normal and Fib Normal); (2) hypertriglyceridemia and normal Fib (TG ↑/Fib Normal); (3) normal TG and hypofibrinogenemia (TG Normal/Fib ↓); and (4) hypertriglyceridemia and hypofibrinogenemia (TG ↑/Fib ↓). The subgroup allocation was shown in Fig. 2.

The demographical and clinical characteristics according to triglyceride-fibrinogen groups was shown in Table 2. The differences of the distribution of sex, age, and underlying diseases were non-significant among three TG-Fib subgroups that defined by the worst values during hospitalization. The rates of hepatic dysfunction, coagulopathy, and respiratory failure were significantly different among three groups. The result of Chi square for trend showed that the rates of MODS were significantly increased across the (TG ↑/Fib Normal), (TG ↑/Fib ↓), and (TG Normal/Fib ↓) groups (27.8%, 58.6%, and 86.2%, respectively, *P* for trend < 0.0001); and the mortality rates also increased across the (TG ↑/Fib Normal), (TG ↑/Fib ↓), and (TG Normal/Fib ↓) groups (18.8%, 24.3%, and 57.1%, respectively, *P* for trend = 0.0115). The subgroups defined by the worst values of TG and Fib within 72 h of hospitalization showed increasing trends of MODS and mortality among (TG ↑ and Fib Normal), (TG Normal and Fib Normal), (TG ↑ and Fib ↓), and (TG Normal and Fib ↓) groups (*P* values for trend < 0.05).

**Table 2:** Comparison of demographical and clinical characteristics according to triglyceride-fibrinogen groups

Characteristics	All	Triglyceride- fibrinogen groups				*P*
		TG:↑ Fib: Normal	TG: Normal Fib: Normal	TG: ↑ Fib: ↓	TG: Normal Fib:↓	
Grouping according to worst values during hospitalization						
Total	117	18	0	70	29	
Sex						
Male	65 (55.6)	10 (55.6)	0	37 (52.9)	18 (62.1)	0.7030
Female	52 (44.4)	8 (44.4)	0	33 (47.1)	11 (37.9)	
Age						
≤ 12 months	17 (14.5)	1 (5.6)	0	11 (15.7)	5 (17.2)	0.5743
> 12 months	100 (85.5)	17 (94.4)	0	59 (84.3)	24 (82.8)	
Fulfillment of diagnostic criteria						
Fever	116 (99.1)	18 (100)	0	69 (98.6)	29 (100)	1.0000
Splenomegaly	102 (87.2)	**13 (72.2)**	**0**	**60 (85.7)**	**29 (100)**	**0.0080**
Bicytopenia (≥ 2/3 lineages)	109 (93.2)	**14 (77.8)**	**0**	**67 (95.7)**	**28 (96.6)**	**0.0259**
Hemoglobin < 90 g/L	106 (90.6)	**12 (66.7)**	**0**	**65 (92.9)**	**29 (100)**	**0.0013**
Platelets < 100 × 109 /L	106 (90.6)	**11 (61.1)**	**0**	**67 (95.7)**	**28 (96.6)**	**0.0003**
Neutrophils < 1.0 × 109 /L	76 (65.0)	12 (66.7)	0	48 (68.6)	16 (55.2)	0.4394
Hypertriglyceridemia and/or hypofibrinogenemi	115 (98.3)	17 (94.4)	0	70 (100)	28 (96.6)	0.1593
Triglyceride ≥ 3.0 mmol/L	77 (65.8)	**17 (94.4)**	**0**	**60 (85.7)**	**0**	** < 0.0001**
Fibrinogen ≤ 1.5 g/L	95 (81.2)	**0**	**0**	**67 (95.7)**	**28 (96.6)**	** < 0.0001**
Hemophagocytosis						
Yes	78 (66.7)	11 (61.1)	0	48 (68.6)	19 (65.5)	–^a^
No	34 (29.1)	7 (38.9)	0	19 (27.1)	8 (27.6)	
Missing/not done	5 (4.3)	0	0	3 (4.3)	2 (6.9)	
Low/absent NK-cell activity						–^a^
Yes	82 (70.1)	14 (77.8)	0	50 (71.4)	18 (62.1)	
Missing/not done	35 (29.9)	4 (22.2)	0	20 (28.6)	11 (37.9)	
Ferritin	111 (94.9)	18 (100)	0	66 (94.3)	27 (93.1)	0.7191
Soluble CD25 ≥ 2400 U/ml						
Yes	38 (32.5)	10 (55.6)	0	22 (31.4)	6 (20.7)–	–^a^
No	3 (2.6)	0	0	3 (4.3)	0	
Missing/not done	76 (65.0)	8 (44.4)	0	45 (64.3)	23 (79.3)	
Underlying diseases						
Primary HLH	9 (7.7)	1 (5.6)	0	5 (7.1)	3 (10.3)	0.8840
Autoimmune disorders	4 (3.4)	1 (5.6)	0	2 (2.9)	1 (3.4)	0.7859
Malignancy	6 (5.1)	2 (11.1)	0	4 (5.7)	0	0.1778
Sepsis	75 (64.1)	8 (44.4)	0	46 (65.7)	21 (72.4)	0.1372
EBV infection	80 (68.4)	11 (61.1)	0	52 (74.3)	17 (58.6)	0.2410
Concomitant diagnosis						
Hepatic dysfunction	80 (68.4)	**7 (38.9)**	**0**	**49 (70.0)**	**24 (82.8)**	**0.0064**
Coagulopathy	69 (59.0)	**5 (27.8)**	**0**	**41 (58.6)**	**23 (79.3)**	**0.0022**
DIC	33 (28.2)	2 (11.1)	0	20 (28.6)	11 (37.9)	0.1383
Heart failure	22 (18.8)	2 (11.1)	0	15 (21.4)	5 (17.2)	0.5886
Shock	16 (13.7)	1 (5.6)	0	9 (12.9)	6 (20.7)	0.3472
CNS disease	31 (26.5)	4 (22.2)	0	15 (21.4)	12 (41.4)	0.1113
Acute kidney injury	14 (12.1)	0	0	8 (11.6)	6 (20.7)	0.1068
Respiratory failure	49 (41.9)	**3 (16.7)**	**0**	**30 (42.9)**	**16 (55.2)**	**0.0328**
ARDS	10 (8.5)	1 (5.6)	0	5 (7.1)	4 (13.8)	0.5783
MODS	71 (60.7)	**5 (27.8)**	**0**	**41 (58.6)**	**25 (86.2)**	** < 0.0001**^**b**^
30-day survival^c^						
Survive	78 (68.4)	**13 (81.3)**	**0**	**53 (75.7)**	**12 (42.9)**	**0.0115**^**b**^
Death	36 (31.6)	**3 (18.8)**	**0**	**17 (24.3)**	**16 (57.1)**	
Grouping according to worst values within 72 h of hospitalization						
Available data (n)	89	10	20	34	25	
MODS	71 (60.7)	**3 (30.0)**	**11 (55.0)**	**23 (67.6)**	**19 (76.0)**	**0.0091**^**b**^
30-day survival						
Survive	78 (68.4)	**10 (100)**	**16 (80.0)**	**24 (70.6)**	**12 (50.0)**	**0.0019**^**b**^
Death	36 (31.6)	**0**	**4 (20.0)**	**10 (29.4)**	**12 (50.0)**	

ARDS, acute respiratory distress syndrome; CNS disease, central nervous system disease; DIC, disseminated intravascular coagulation; EBV, Epstein-Barr Virus; HLH, hemophagocytic lymphohistiocytosis; MODS, multiple organ dysfunction syndrome

TG ↑, ≥ 3.0 mmol/L. Fib ↓, ≤ 150 mg/dL

Values in bold are statistically significant (*P* < 0.05)

^a^Between-group comparisons were not conducted due to missing data

^b^*P* for trend (Cochran-Armitage test for trend)

^c^Three patients were lost follow-up at day 30

Table 3 shows the result of Logistic regression analyses that using the (TG ↑ and Fib Normal) group as the reference group to estimate the risk of MODS and 30-day mortality for other TG-Fib groups. For subgroups defined by the worst values during hospitalization, the (TG Normal and Fib ↓) group showed the highest risk of MODS (OR = 16.24, *P* = 0.0002) and 30-day mortality (OR = 5.78, *P* = 0.0187). Similar associations were observed for subgroups derived from the worst values within 72 h (Table 3).

**Table 3:** Risk of MODS and 30-day mortality according to triglyceride-fibrinogen groups

Grouping methods	Triglyceride, mmol/L	Fibrinogen, mg/dL	MODS		30-day mortality	
			OR (95% CI)	*P*	OR (95% CI)	*P*
According to worst values during hospitalization	↑, ≥ 3.0	Normal	1 (Reference)		1 (Reference)	
	Normal	Normal	–		–	
	↑, ≥ 3.0	↓, ≤ 150	**3.68 (1.18, 11.44)**	**0.0247**	1.39 (0.35, 5.46)	0.6374
	Normal	↓, ≤ 150	**16.24 (3.71, 71.05)**	**0.0002**	**5.78 (1.34, 24.92)**	**0.0187**
According to worst values within 72 h	↑, ≥ 3.0	Normal	1 (Reference)		–	
	Normal	Normal	2.85 (0.57, 14.33)	0.2032	1 (Reference)	
	↑, ≥ 3.0	↓, ≤ 150	**4.88 (1.05, 22.57)**	**0.0425**	1.67 (0.44, 6.24)	0.4485
	Normal	↓, ≤ 150	**7.39 (1.44, 37.88)**	**0.0165**	**4 (1.03, 15.53)**	**0.0452**

HLH, hemophagocytic lymphohistiocytosis; MODS, multiple organ dysfunction syndrome

Values in bold are statistically significant (*P* < 0.05)

Additional analyses were conducted to examine the changing of triglyceride and fibrinogen value status from the first 72 h to later period of hospitalization and its association with clinical outcomes (Additional file 1: Table S3). Patients who reported normal TG within 72 h of hospitalization and developed hypertriglyceridemia later showed lower rates of MODS than that of patients who reported normal TG during hospitalization (52% and 86.4%, respectively; *P* = 0.0117). Patients who presented normal FIB within 72 h and developed hypofibrinogenemia later showed a higher rate of MODS than that of patients with normal Fib during hospitalization (63.5% and 26.7%, respectively; *P* = 0.0272).

## Discussion

### Main findings

Triglyceride and fibrinogen were identified as class defining variables. Hypofibrinogenemia and transforming form normal fibrinogen level to hypofibrinogenemia during hospitalization were associated with higher risks of MODS and 30-day mortality. Hypertriglyceridemia and transforming form normal triglyceride level to hypertriglyceridemia during hospitalization were associated with lower risks of MODS and 30-day mortality. When triglyceride and fibrinogen were evaluated in combinations, patients with hypertriglyceridemia and normal fibrinogen levels had the lowest risks for adverse outcomes, and patients with normal triglyceride and hypofibrinogenemia had the highest risks for adverse outcomes; these associations were statistically significant both within 72 h of hospital admission and during hospitalization.

### Implication

The complexity of clinical presentations and triggering diseases add difficulties to the diagnosis of HLH. Based on the current understanding of HLH pathogenesis, the features of patients with HLH were categorized into three categories [2]: predisposing immunodeficiency (e.g. low or absent NK-cell function and genetic defect of cytotoxicity), significant immune activation (e.g. fever, splenomegaly, elevated ferritin, and elevated sCD25), and abnormal immunopathology (e.g. cytopenias, decreased fibrinogen or increased triglycerides, hemophagocytosis). Not all of the HLH patients met all of the HLH diagnostic criteria, and not all of the features presented initially [12]. This research started from the question that how the fulfillment of HLH diagnostic criteria associated with disease severity. We used a data driven approach to identify the class-defining criteria from 9 criteria which were routinely available, and tested whether the combinations of different fulfillment status of class-defining criteria were associated with disease severity and outcomes. The LCA analysis identified triglyceride and fibrinogen as the class-defining variables, and the classification method using triglyceride-fibrinogen combinations showed promising result on the identification of patients at higher or lower risks for MODS and 30-day mortality.

Hypertriglyceridemia in HLH is thought to be secondary to decreased lipoprotein lipase activity, which was initiated by increased TNF-α levels [13]. Few studies investigated the association between hypertriglyceridemia and HLH outcomes among children, and these studies observed non-significant associations [7, 8, 14]. Different from previous studies, our study observed a negative association between hypertriglyceridemia and adverse outcomes. Several factors could lead to the inconsistent findings, including the sampling time (at hospital admission, at diagnosis, or worst value during hospitalization), severity of disease, and the distribution of underlying diseases and the unrevealed disease subphenotypes. As few comparable studies were available, we need more evidence to come to a conclusion. Notably, the protective effect of hypertriglyceridemia on disease outcomes was reported in sepsis [15, 16]. The triglyceride-rich lipoproteins were thought to be components of an innate, non-adaptive host immune response to infection [15, 16]. It was conceptualized that hepatic metabolism of triglyceride-rich lipoproteins is part of a cytokine-mediated host homeostatic mechanism; during the acute-phase response, the cytokine-mediated change in gene expression resulted in the increased production and release of triglyceride-rich lipoproteins [16]. The findings and theories from the studies of sepsis might provide a plausible explanation for the protective effect of triglyceride in HLH, as HLH is characterized by cytokine storm and overwhelming inflammation, and the shared disease manifestation between sepsis and HLH implies the potential overlap between their etiology [17, 18]. After all, more studies are needed to validate our finding and explore the role of triglyceride in the pathology of HLH.

Activated macrophages may secrete plasminogen activators which accelerate the conversion of plasminogen to plasmin, leading to the degradation of fibrinogen [13]. Hypofibrinogenemia were found to be negatively associated with survival in both pediatric [7] and adult [19] HLH patients. Although non-significant association between hypofibrinogenemia and adverse outcomes has also been reported [14]. As mentioned above, many factors could affect the observed associations. Several studies did not report when the blood samples were obtained, thus limiting the comparability between studies. Our study investigated the worst value of fibrinogen within 72 h or hospital admission and during hospitalization, and found that the presence of hypofibrinogenemia during hospitalization was associated with higher risks of MODS and 30-day mortality. Besides, compared with patients with normal fibrinogen levels during hospitalization, these patients transforming form normal fibrinogen level into hypofibrinogenemia showed higher rates of MODS and 30-day mortality. Although further studies are needed to validate these findings, our data demonstrated the potential utility of this newly identified class-defining variable.

It is believed that HLH comprises a heterogeneous spectrum of clinically similar but etiologically diverse subtypes, however, few studies explored the subtypes of HLH, possibly because it is a relatively rare disease, whereas the data driven approach for complex disease usually require large sample size. For example, in the searching of subphenotypes for sepsis and ARDS, studies usually included more than thousands of cases [20, 21]. Generally, the more variables included in a model, the larger of the sample size will be needed to build a robust model. As an alternative strategy, rather than directly using the classification model derived from the LCA, we only used the LCA to identify the class defining variables, and then used the simple combinations of these variables for subgrouping. This approach was intend to avoid overfitting of the LCA model, and to reduce the model variables to increase the easiness of its application.

Our results showed that the combination of triglyceride and fibrinogen fulfillment status could be used to stratify HLH patients into lower or higher risk groups for MODS and 30 day mortality. These findings partially answered our study question that how the fulfillment of HLH diagnostic criteria could be used to evaluate disease severity. However, this is the first study reported the association between the triglyceride-fibrinogen combinations and HLH severity/outcome, clinical studies in other centers and in multiple populations are needed to investigate whether this method could be used for the clinical evaluation of disease severity, which is now lacking in the clinical practice of HLH [22, 23]. The mechanism underlying the observed associations also worth further investigation, which might provide us with better understanding of the disease pathophysiology and trajectory. Furthermore, studies might investigate whether this risk stratification method could be used in the targeted treatment of HLH; as the immunosuppressive and cytotoxic effect of chemotherapy for severe HLH could induce severe side-effects, how to distinguish HLH patients need aggressive treatment from patients who could remit with less aggressive treatment is a clinical challenge [22, 23].

The strengths of this study including it for the first time reported the association between combination of items from the HLH diagnostic criteria and the risk of MODS and 30-day mortality among pediatric patients; these findings might promote our understanding of the clinical relevance of the fulfillment of diagnostic criteria, and might provide a simple method to evaluate the severity of HLH use easily available tests. Besides, the values of triglyceride and fibrinogen within 72 h and during hospitalization, as well as the changes of their status were investigated, all showed consistent associations with HLH severity and outcomes, demonstrating the potential capacity of this method to be used soon after hospital presentation and during hospitalization.

This study had several limitations. First, it is a single center study. The validity and generalizability of its findings must be further investigated. As HLH is a heterogeneous, and a relatively rare disease, multicenter studies included multiple populations are needed before the application of the new evaluation method. Second, this study only used routinely available clinical data from the medical records, limiting our ability to describe the pathological features of different subgroups. Third, the sample sized of this study is relatively small for the purpose of finding subgroups. Fourth, there is very little information regarding the explanation for the observed association between triglyceride and HLH outcomes. Further exploration is necessary to investigate not only the clinical utility of the combined evaluation of triglyceride and fibrinogen, but also the underlying pathological mechanism.

To conclude, when evaluated in combinations, the fulfillment status of hypertriglyceridemia and hypofibrinogenemia were associated with the risks of MODS and 30-day mortality among pediatric HLH patients. Further studies are needed to validate this association and investigate its clinical utility in the severity evaluation for HLH.

## Methods

### Study population

This study retrospectively included pediatric HLH patients (age ≤ 18 years old at the time of HLH diagnosis) that discharged from Hunan Children's Hospital in China between June 2015 and October 2018. The chart review was conducted between May 2019 and August 2021. The exclusion criteria included patients diagnosed with HLH prior to the indicated hospital admission date, and patients with essential diagnostic/subgrouping data missing.

The study protocol was reviewed and approved by the Medical Ethics Committee of the Hunan Children's Hospital (HCHLL-2019-40) and have been performed in accordance with the ethical standards as laid down in the 1964 Declaration of Helsinki and its later amendments or comparable ethical standards. The requirement for written informed consent was waived by the Medical Ethics Committee of the Hunan Children's Hospital.

### Variables and diagnostic criteria

HLH was diagnosed in accordance with the HLH-2004 criteria [1]. According to HLH-2004 diagnostic criteria, the diagnosis of HLH can be established if one of either A or B below is fulfilled [1]: A. A molecular diagnosis consistent with HLH. B. Five out of eight criteria fulfilled: (1) Fever. (2) Splenomegaly. (3) Cytopenias affecting 2 of 3 lineages (hemoglobin < 90 g/L, platelets < 100 × 10^9^/L, and/or neutrophils < 1.0 × 10^9^/L) in the peripheral blood. (4) Hypertriglyceridemia and/or hypofibrinogenemia: fasting triglycerides ≥ 3.0 mmol/L, fibrinogen ≤ 1.5 g/L. (5) Hemophagocytosis in bone marrow or spleen or lymph nodes. No evidence of malignancy. (6) Low or absent NK-cell activity. (7) Ferritin ≥ 500 μg/L. (8) Soluble CD25 (sCD25) ≥ 2400 U/ml. Primary HLH was diagnosed based on a family history of HLH and/or a molecular (genetic) diagnosis of HLH.

Nine candidate class-defining variables were selected from the diagnostic criteria, including fever, splenomegaly, hemoglobin, platelets, neutrophils, triglycerides, fibrinogen, hemophagocytosis, and ferritin. Genetic test result, NK-cell activity, and sCD25 were not routine practices in the study center during the study period, and a large proportion of patients had missing value for these tests, therefore these tests were excluded from the candidate variables. The worst value for each candidates were dichotomized as normal and abnormal according to the diagnostic criteria.

The main outcome variable was 30-day mortality. The secondary outcome was MODS during hospitalization. Other demographical and clinical variables included: age, sex, underlying diseases (primary HLH, autoimmune disorders, malignancy, sepsis [24], and EBV infection), and concomitant diagnoses, including hepatic dysfunction [25], coagulopathy, disseminated intravascular coagulation (DIC), heart failure [26], shock [24, 27], central nervous system disease (CNS) disease, acute kidney injury [28], respiratory failure, and acute respiratory distress syndrome (ARDS) [29].

### Statistical analysis

Latent class analyses were conducted by SAS 9.4 (SAS Institute, Inc., Cary, NC) using the PROC LCA step. In the latent class analyses, we built three models consisting of two, three, and four classes, respectively. Optimal number of classes was evaluated using a combination of entropy, Bayesian information criteria (BIC), and Akaike information criteria (AIC). Entropy, ranges from zero to one, is an index of how well the classes are separated. A entropy values 0.8 or higher is thought to be a sign of good class separation. Decreasing values in BIC or AIC indicate improved model fit. Once the optimal number of classes was identified, the class membership probabilities for each model-defining variable were exported. For each variable, the differences in probabilities for each class were calculated. Variables with the largest between-class probability differences were identified as class defining variables.

Data are presented as absolute values and percentages, or median, range and quartiles (Q1 and Q3), as appropriate. Between-group comparisons for categorical variables were conducted using chi-squared test or Fisher's exact test, as appropriate. Chi square test for trend (Cochran-Armitage test for trend) was used to test the trend of the associations between subgroups and risk of 30-day mortality and MODS. Between-group comparisons for continuous variables were conducted by Wilcoxon rank sum test. Logistic regression analyses were used to estimate the association between subgroups and adverse outcomes, using the group with the lowest risk as the reference group. All tests were set two-tailed with a type 1 error rate fixed at 5%. Missing data was not imputed. All statistical analyses were performed using SAS 9.4 (SAS Institute, Inc., Cary, NC).

## Supplementary Information

**Additional file 1: Table S1**. Fit statistics for latent class models from one to four latent classes. **Table S2**. Class probability for model-defining variables. **Table S3**. Changing of triglyceride and fibrinogen value status and clinical outcomes among pediatric HLH patients

## Data Availability

The datasets used and/or analysed during the current study are available from the corresponding author on reasonable request.

## Abbreviations

AIC: Akaike information criteria
ARDS: Acute respiratory distress syndrome
BIC: Bayesian information criteria
CNS disease: Central nervous system disease
DIC: Disseminated intravascular coagulation
EBV: Epstein-Barr Virus
Fib: Fibrinogen
HLH: Hemophagocytic lymphohistiocytosis
LCA: Latent class analysis
MODS: Multiple organ dysfunction syndrome
OR: Odds ratio
sCD25: Soluble CD25
TG: Triglyceride
