# Supplementary material for: Fulfillment status of hypertriglyceridemia and hypofibrinogenemia in children with hemophagocytic lymphohistiocytosis and risks of multiple organ dysfunction syndrome and early mortality
Source: Orphanet J Rare Dis. 2022 Apr 11;17:161. doi: 10.1186/s13023-022-02315-8 (PMC8996201; doi:10.1186/s13023-022-02315-8)
Supplement: Supplementary file 1 — Additional file 1: Table S1. Fit statistics for latent class models from one to four latent classes. Table S2. Class probability for model-defining variables. Table S3. Changing of triglyceride and fibrinogen value status and clinical outcomes among pediatric HLH patients [file 13023_2022_2315_MOESM1_ESM.docx]

Additional file 1: Table S1. Fit statistics for latent class models from one to four latent classes

| No. of classes | Entropy | AIC | BIC |
| --- | --- | --- | --- |
|  |  |  |  |
| 2 | 1 | 91.03 | 143.51 |
| 3 | 0.98 | 103.83 | 183.94 |
| 4 | 0.81 | 104.49 | 212.21 |

AIC, Akaike information criteria; BIC, Bayesian information criteria.

Additional file 1: Table S2. Class probability for model-defining variables

| Variables (value =abnormal) | Class 1 | Class 2 | Difference |
| --- | --- | --- | --- |
| Fibrinogen | 1 | 5.63E-06 | 0.999994 |
| Platelets | 0.979798 | 0.777778 | 0.20202 |
| Ferritin | 0.804348 | 0.625 | 0.179348 |
| Hemoglobin | 0.989899 | 0.888889 | 0.10101 |
| Splenomegaly | 0.929293 | 0.833334 | 0.095959 |
| Neutrophils | 0.868687 | 0.777778 | 0.090909 |
| Fever | 0.989899 | 1 | -0.0101 |
| Hemophagocytosis | 0.919192 | 1 | -0.08081 |
| Triglyceride | 0.70707 | 1 | -0.29293 |

Additional file 1: Table S3. Changing of triglyceride and fibrinogen value status and clinical outcomes among pediatric HLH patients

| Changing of worst values status | | n | MODS | | |  | 30-day survival | | |
| --- | --- | --- | --- | --- | --- | --- | --- | --- | --- |
| Within 72 hours | During hospitalization |  | No | Yes | *P* |  | Survive | Death | *P* |
| TG: Normal | TG: Normal | 22 | **3 (13.6)** | **19 (86.4)** | **0.0117** |  | 11 (52.4) | 10 (47.6) | 0.0679 |
|  | TG:↑ | 25 | **12 (48.0)** | **13 (52.0)** |  |  | 6 (24.0) | 19 (76.0) |  |
|  |  |  |  |  |  |  |  |  |  |
| TG:↑ | TG:↑ | 45 | 19 (42.2) | 26 (57.8) |  |  | 10 (22.2) | 35 (77.8) |  |
|  |  |  |  |  |  |  |  |  |  |
| Fib: Normal | Fib: Normal | 15 | **11 (73.3)** | **4 (26.7)** | **0.0272** |  | 3 (23.1) | 10 (76.9) | 1 |
|  | Fib:↓ | 22 | **8 (36.4)** | **14 (63.6)** |  |  | 4 (18.2) | 18 (81.8) |  |
|  |  |  |  |  |  |  |  |  |  |
| Fib:↓ | Fib:↓ | 69 (100) | 21 (30.4) | 48 (69.6) |  |  | 27 (39.7) | 41 (60.3) |  |

HLH= hemophagocytic lymphohistiocytosis. MODS= multiple organ dysfunction syndrome.

TG ↑, ≥3.0 mmol/L. Fib ↓, ≤150 mg/dL.

Values in bold are statistically significant (*P*<0.05).
